# Mixture-Based Probabilistic Graphical Models for the Label Ranking Problem †

**DOI:** 10.3390/e23040420

**Published:** 2021-03-31

**Authors:** Enrique G. Rodrigo, Juan C. Alfaro, Juan A. Aledo, José A. Gámez

**Affiliations:** 1 Departamento de Sistemas Informáticos, Universidad de Castilla-La Mancha, 02071 Albacete, Spain; mail@enriquegrodrigo.com (E.G.R.); Jose.Gamez@uclm.es (J.A.G.); 2 Laboratorio de Sistemas Inteligentes y Minería de Datos, Instituto de Investigación en Informática de Albacete, 02071 Albacete, Spain; JuanAngel.Aledo@uclm.es; 3 Departamento de Matemáticas, Universidad de Castilla-La Mancha, 02071 Albacete, Spain

**Keywords:** mixture models, EM algorithm, Naive Bayes, probabilistic graphical models, label ranking, preference learning, machine learning

## Abstract

The goal of the *Label Ranking (LR) problem* is to learn *preference models* that predict the preferred ranking of class labels for a given unlabeled instance. Different well-known machine learning algorithms have been adapted to deal with the LR problem. In particular, fine-tuned instance-based algorithms (e.g., k-nearest neighbors) and model-based algorithms (e.g., decision trees) have performed remarkably well in tackling the LR problem. *Probabilistic Graphical Models* (*PGMs*, e.g., *Bayesian networks*) have not been considered to deal with this problem because of the difficulty of modeling permutations in that framework. In this paper, we propose a *Hidden Naive Bayes classifier* (*HNB*) to cope with the LR problem. By introducing a hidden variable, we can design a hybrid Bayesian network in which several types of distributions can be combined: multinomial for discrete variables, Gaussian for numerical variables, and *Mallows* for permutations. We consider two kinds of probabilistic models: one based on a *Naive Bayes* graphical structure (where only univariate probability distributions are estimated for each state of the hidden variable) and another where we allow interactions among the predictive attributes (using a multivariate Gaussian distribution for the parameter estimation). The experimental evaluation shows that our proposals are competitive with the start-of-the-art algorithms in both accuracy and in CPU time requirements.

## 1. Introduction

Preferences are comparative judgments about a set of alternatives or choices. The *Label Ranking (LR) problem* [1,2,3] is a well-known non-standard supervised classification problem [4,5], whose goal is to learn *preference models* that predict the preferred ranking over a set of class labels for a given unlabeled instance. Practical applications of the LR problem are found in cases where an order of preference (or ranking) for the class labels is required given an input instance. Particular examples can be ranking a set of genes from their expression level, ranking the set of relevant topics for a given document, ranking a set of available machine learning algorithms for a given dataset and prediction task, etc. [6,7].

Formally, we consider a problem domain defined over *npredictive variables* (also known as *attributes*), $X_{1},\ldots ,X_{n}$, and a *class variableC* with *m* labels, $dom\left(C\right)=\left\{c_{1},\ldots ,c_{m}\right\}$. We are interested in predicting the ranking π of the labels for an unlabeled instance $e_{t}=\left(x_{1,t},\ldots ,x_{n,t}\right)\in dom\left(X_{1}\right)\times \ldots \times dom\left(X_{n}\right)$ given a dataset $D={\left\{\left(x_{1,j},\ldots ,x_{n,j},\pi_{j}\right)\right\}}_{j=1}^{N}$ with *N* labeled instances. Therefore, the LR problem consists in learning a LR-Classifier C from D which generalizes well on unseen data.

In other words, the goal of the LR problem is to induce a model able to predict a permutation of the class labels by taking advantage of all the available information during the learning process. Different approaches have been proposed to tackle this problem:

- *Transformation methods*. They transform the ranking-based prediction problem into a set of single-class classifiers, whose outcomes must be later aggregated in order to obtain a ranking. Various approaches have been considered, such as *labelwise* [8] and *pairwise* approaches [9,10], *chain classifiers* [11], etc.
- *Adaptation methods*. They adapt well-known machine learning algorithms to cope with the new class structure. Cheng et al. in [2] introduced a *model-based algorithm* that induces a decision tree (*Label Ranking Trees (LRT)*) and a *model-free algorithm* which uses *k*-nearest neighbors (*Instance-Based Label Ranking (IBLR)*). Other techniques, like association rules [12] or neural networks [13], have also been adapted.
- *Ensemble methods*. Recently, different tree-based aggregation approaches, such as *Random Forests*, *Bagging predictors*, and *Boosting methods*, have been successfully applied to the LR problem [14,15,16,17].

In this paper, we propose a new model-based LR-classifier which belongs to the adaptation methods family. Our motivation is twofold:

- Although fine-tuned instance-based algorithms have exhibited remarkable performance (especially when the model is trained with *complete rankings*), they may demand a great amount of computational resources (memory and time) during model selection and inference when the size of the dataset grows.
- Although *Probabilistic Graphical Models* (*PGMs*; e.g., *Bayesian networks*) [18,19] constitute a standard approach in machine learning, they have not been used in this problem because of the difficulty in coping with permutations in this framework [2,20]. In this work, we successfully introduce the use of PGMs to deal with the LR problem, obtaining results which are competitive with the state-of-the-art IBLR and LRT algorithms.

The proposed probabilistic LR-classifier relies on the use of a *hybrid Bayesian network* [21] where different probability distributions are used to conveniently model variables of a different nature: Multinomial for discrete variables, Gaussian for numerical variables, and *Mallows* for permutations [22]. The Mallows probability distribution is usually considered to model a set of permutations and, in fact, is the core of the decision tree algorithm (LRT) proposed in [2].

To overcome the constraints regarding the topology of the network when dealing with different types of variables, in the preliminary version of this study, we proposed a mixture-based structure where the root is a *hidden discrete variable*. In [23], we based our proposal on a *Naive Bayes* graphical structure, where only univariate probability distributions are estimated for each state of the hidden variable. Learning and inference schemes were also designed in [23], based on the use of well-known *Expectation-Maximization* (EM) algorithm for parameter estimation and a combination of probabilistic inference with the *Kemeny Ranking Problem* (*KRP*) [24], respectively. Nonetheless, the proposed methods performed somewhat unevenly when dealing with the different datasets. With this more comprehensive paper, we successfully overcome the main weaknesses of our former proposal. Specifically, the main contributions of this study are as follows:

- After identifying early stopping as the main problem in our previous learning algorithm (Method A), we propose a new learning scheme (see Method B in Section 3) to search for the number of components in the mixture.
- For our Hidden Naive Bayes model, we explore discretization as an alternative to modeling numerical variables as Gaussian distributions.
- We extend the complexity of the naive Bayes-based structure model in order to allow interactions among the predictive attributes. In this new model, only numerical predictive attributes are allowed, and interactions are managed by using a multivariate Gaussian distribution.
- We perform an exhaustive experimental analysis over the standard benchmark for the label ranking problem.

The rest of the paper is structured as follows. In Section 2, we review some basic notions needed to deal with rank data. In Section 3, we formally describe the proposed *Hidden Naive Bayes* (*HNB*) as well as the algorithms to induce it from data and to carry out inference. Then, in Section 4, we extend our proposal to allow interactions between the (numerical) predictive attributes, by using a multivariate Gaussian mixture. In Section 5, we set out the empirical study conducted to evaluate the methods designed in this paper. In Section 6, we briefly comment on some limitations of the presented approach. Finally, in Section 7, we provide the conclusions and future research lines.

## 2. Background

In this section, we review the background to our proposal. In particular, we briefly describe some permutation-based notions, such as the *Kemeny Ranking Problem* [24] and the *Mallows probability distribution* [22]. We also revise the *Naive Bayes* model [18] and the two competing methods to tackle the LR problem used in this study: the *Label Ranking Trees* and the *Instance-Based Label Ranking* algorithms [2].

### 2.1. Kemeny Ranking Problem

Let $S_{m}$ be the set of permutations defined over *m* elements {1,…,m}. The *Kemeny Ranking Problem* (*KRP*) [24] consists in obtaining the *consensus permutation* (*mode*) $\pi_{0}\in S_{m}$ that best represents a sample with *N* permutations $\Pi =\{\pi_{1},\ldots ,\pi_{N}\}$, $\pi_{i}\in S_{m}$.

Formally, the KRP looks for the consensus permutation $\pi_{0}\in S_{m}$ that minimizes

$$\pi_{0}=\underset{\pi_{i}\in S_{m}}{argmin}\sum_{i=1}^{N}D\left(\pi_{0},\pi_{i}\right),$$

where D(π,τ), $\pi ,\tau \in S_{m}$ is a distance measure between two permutations π and τ. Normally, the *Kendall distance* [25] is used, which counts the number of pairwise disagreements between the two permutations, and the (greedy) *Borda count* algorithm [26] is employed to solve the KRP, because of its trade-off between efficiency and accuracy. The Borda count algorithm basically assigns *m* points to the item ranked first, m−1 to the second one, and so on. Once all the input rankings have been computed, the items are sorted according to the number of accumulated points.

When not all rankings are equally important, a weight can be associated with each one to reflect its relevance. Then, a generalized version of the Borda method called *weighted Borda count* is used, which basically balances the points received by a permutation taking its weight into account.

### 2.2. Kendall Rank Correlation Coefficient

In our learning process (see Section 3.3), the *Kendall rank correlation coefficient* $\tau_{K}$ is used as goodness score [27]. Given the class variable *C* with $dom\left(C\right)=\left\{c_{1},\ldots ,c_{m}\right\}$ and permutations $\pi_{1},\pi_{2}$ of the values in dom(C), the $\tau_{K}$ Kendall rank correlation coefficient is given by

$$\tau_{K}\left(\pi_{1},\pi_{2}\right)=\frac{\sum_{i=1}^{m}\sum_{j=1}^{m}\beta_{1}^{ij}\cdot \beta_{2}^{ij}}{m\cdot (m-1)}$$

where

$$\beta_{k}^{ij}=\left\{\begin{array}{cc} 1, & \mathrm{if}\;c_{i}{≻}_{\pi_{k}}c_{j}\\ -1, & \mathrm{if}\;c_{j}{≻}_{\pi_{k}}c_{i}\\ 0, & \mathrm{if}\;i=j \end{array}\right.$$

for k=1,2. Here, $c_{i}{≻}_{\pi_{k}}c_{j}$ means that $c_{i}$ is ranked before $c_{j}$ in $\pi_{k}$.

The $\tau_{K}$ Kendall rank correlation coefficient lies in the range [−1,1]. In particular, $\tau_{K}\left(\pi_{1},\pi_{2}\right)=1$ means a total positive correlation between $\pi_{1}$ and $\pi_{2}$ ($\pi_{1}=\pi_{2}$), whereas $\tau_{K}\left(\pi_{1},\pi_{2}\right)=-1$ indicates a total negative correlation (actually this occurs when $\pi_{1}$ is the inverse of $\pi_{2}$). Values of $\tau_{K}$ close to 0 mean a poor correlation between the permutations.

### 2.3. Mallows Probability Distribution

The *Mallows probability distribution* (also known as the *Mallows model*) [22] is an exponential probability distribution over permutations based on distances. The Mallows model, $M(\pi_{0},\theta )$, is parametrized by two parameters: the *central permutation* (*mode*) $\pi_{0}\in S_{m}$ and the *spread parameter* (*dispersion*) θ∈[0,+∞). Given a distance *D* in $S_{m}$, the probability assigned to a permutation $\pi \in S_{m}$ by the Mallows distribution $M(\pi_{0},\theta )$ is

$$P\left(\pi ;\pi_{0},\theta \right)=\frac{e^{-\theta \cdot D(\pi ,\pi_{0})}}{\Psi (\theta )}$$

where Ψ(θ) is a normalization constant. The spread parameter θ quantifies the concentration of the distribution around $\pi_{0}$. For θ=0, a uniform distribution is obtained, while for θ=+∞ the model assigns a probability of 1 to $\pi_{0}$ and of 0 to the rest of the permutations. Both $\pi_{0}$ and θ can be estimated accurately in polynomial time [28]. For consensus permutation ($\pi_{0}$), the Borda count is usually employed. For the spread (θ), there is no closed form, so numerical algorithms (e.g., Newton–Raphson) are normally used.

In this study, we take the Kendall distance as *D*, which is the usual choice in the literature [2,29].

### 2.4. Naive Bayes

**Figure 1:**
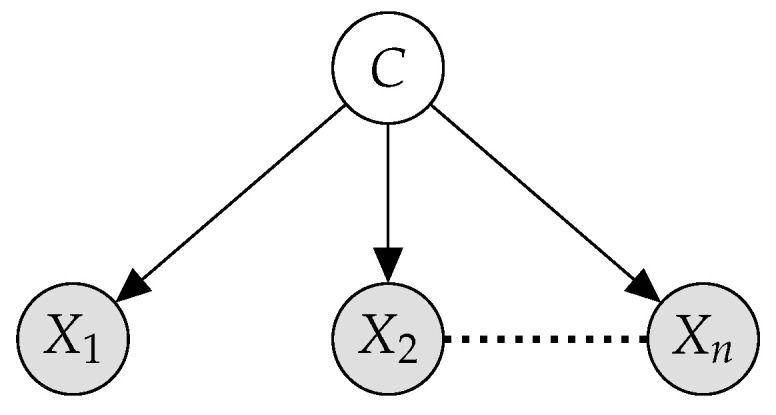
Naive Bayes model structure.

*Naive Bayes* (*NB*) models are well-known *probabilistic classifiers* based on the strong independence hypothesis that, given the class variable, every pair of features is considered conditionally independent [30]. This assumption allows an efficient factorization of the join probability distribution (see Equation (1)) as well as efficient learning and inference procedures. Figure 1 shows the graphical structure of an NB model.

Like most probabilistic classifiers, NB models follow the *maximum a posteriori* (*MAP*) principle, that is, they return the most probable class label given the input instance as evidence. Formally, given an input instance $e_{t}=\left(x_{1,t},\ldots ,x_{n,t}\right)\in dom\left(X_{1}\right)\times \ldots \times dom\left(X_{n}\right)$ and being *C* the class variable with $dom\left(C\right)=\left\{c_{1},\ldots ,c_{m}\right\}$, a *Naive Bayes Classifier* C returns

(1) $$C\left(e_{t}\right)=\underset{c\in dom(C)}{argmax}P\left(c\;|e_{t}\right)=\underset{c\in dom(C)}{argmax}P\left(e_{t},c\right)=\underset{c\in dom(C)}{argmax}\prod_{i=1}^{n}P\left(x_{i,t}|c\right)\cdot P\left(c\right)$$

according to *Bayes’ theorem* and the conditional independence hypothesis, respectively. The above conditional distributions may be multinomial for discrete attributes and Gaussian for continuous attributes.

### 2.5. Instance-Based Label Ranking

The *Instance-Based Label Ranking* (*IBLR*) algorithm [2] is based on the nearest neighbors estimation principle. It takes, as input, an instance $e_{t}$ to be classified, a training dataset D with *N* labeled instances and the number of nearest neighbors $k\in N^{+}$, k≤N, to be considered. Using an appropriate distance, the IBLR algorithm then compares the input instance with all the *N* training ones, obtains the *k* nearest neighbors from D, $R={\left\{\left(x_{1,j},\ldots ,x_{n,j},\pi_{j}\right)\right\}}_{j=1}^{k}$, and takes the rankings associated with these instances, $R_{\Pi }={\left\{\pi_{j}\right\}}_{j=1}^{k}$. Then, the IBLR algorithm applies the Borda count algorithm to the permutations in $R_{\Pi }$ and the obtained consensus permutation $\pi_{0}$ is returned as output.

The main advantage of instance-based learning is its local behavior, which allows it to locally estimate a different target function for each new instance to be classified instead of estimating a single target function for the entire instance space. On the other hand, its main disadvantage is its high computational cost in the inference stage, as it must compare the input instance against all the instances in the training dataset.

### 2.6. Label Ranking Trees

Decision trees are usually constructed by recursively partitioning the dataset. The *Label Ranking Trees* (*LRT*) algorithm [2] receives, at each call, a set of instances $R={\left\{\left(x_{1,j},\ldots ,x_{k,j},\pi_{j}\right)\right\}}_{j=1}^{s}$ with 1≤k≤n and 2≤s≤N, and must decide whether to stop the recursive call by creating a leaf node, or go on with the branching process by splitting the received training dataset R into several subsets according to the value of an attribute $X_{i}$.

The stopping and splitting criteria used in LRT are as follows:

- *Stopping criterion*. If we consider $R_{\Pi }={\left\{\pi_{j}\right\}}_{j=1}^{s}$ as the rankings associated with the instances in R, the LRT algorithm stops the splitting process and creates a leaf node if either of the following two conditions hold:
  - All the rankings are consistent. For all the pairs of class labels $c_{u},c_{v}\in dom\left(C\right)$, they maintain the same preference relation $(c_{u}≻c_{v}$ or $c_{v}≻c_{u})$ through all the rankings in $R_{\Pi }$ which rank both $c_{u}$ and $c_{v}$.
  - s<2m. This condition is introduced as a pre-pruning operation to prevent overfitting.
  The leaf created is labeled with the consensus ranking $\pi_{0}$ obtained by applying the Borda count algorithm over the rankings in $R_{\Pi }$.
- *Splitting criterion*. The LRT algorithm uses the spread parameter θ of the Mallows model (see Section 2.3) to measure the scattering of the rankings associated with a partition with respect to the consensus one. Formally, given an attribute $X_{i}$ with domain $dom\left(X_{i}\right)=\left\{x_{1},\ldots ,x_{r_{i}}\right\}$, the uncertainty associated with a partition $\left\{R_{1},R_{2},\ldots ,R_{r_{i}}\right\}$ of R is inversely proportional to
  (2) $$f\left(X_{i}\right)=f\left(R_{1},\ldots ,R_{r_{i}}\right\})=\sum_{j=1}^{r_{i}}\frac{\left|R_{j}\right|\cdot \theta_{j}}{\left|R\right|},$$
  where $\theta_{j}$ is the spread parameter estimated from the rankings of the instances in $R_{j}$, which can be computed by means of standard numerical optimization methods [2,29].
  The LRT algorithm proceeds in a standard way, that is, sorting the values of the attributes $X_{i}$ in R and analyzing all the possible thresholds λ. Thus, it deals with the resulting two-state discrete attribute $X_{i}^{\lambda }$ with domain $dom\left(X_{i}^{\lambda }\right)=\left\{X_{i}\leq \lambda ,X_{i}>\lambda \right\}$, and selects the threshold λ of the attribute $X_{i}$ that maximizes (2).

Then, an instance is classified by following the path from the root to the corresponding leaf, selecting, at each decision node, the branch corresponding to the value of the attribute in the instance to be classified. Thus, once a leaf node is reached, the permutation assigned to the leaf node is returned.

## 3. Hidden Naive Bayes for Label Ranking

In this section, we propose an NB-based model to deal with the LR problem. We start by defining the proposed PGM structure and then describe the parameter estimation process and two different methods for training the model.

### 3.1. Model Definition

To overcome the constraints regarding the topology of the network when dealing with different types of variables, the model proposed here combines an NB structure with a hidden (latent) variable.

This idea is not new, and has been used, for instance, for unsupervised clustering [21,31], to improve the performance (accuracy) of the base classifier [32], relax some of the independence statements increasing the classifier modeling capability [33,34,35], or obtain models for efficient probabilistic inference [36].

In this paper, the introduction of the hidden variable stems from the need to model the join probability distribution involving variables of a different nature: discrete, continuous, and permutation-based.

In the proposed NB model graphical structure, the root element of the model is a discrete hidden variable, which we will denote as *H*, with $dom\left(H\right)=\left\{h_{1},\ldots ,h_{r_{H}}\right\},r_{H}$ being the total number of mixture models. The rest of the variables are observed variables. We consider two types of observed variables:

- The *feature variables*, observed both in the training and in the test phase. We consider two kinds: discrete variables, denoted as $X_{j}$, $j=1,\ldots ,n_{J}$ and $dom\left(X_{j}\right)=\left\{x_{j_{1}},\ldots ,x_{j_{r_{j}}}\right\}$, and continuous variables, denoted as $Y_{k}$, $k=1,\ldots ,n_{K}$.
- The *ranking variable*, denoted as π, which takes values in $S_{m}$, this being the set of permutations defined over the class labels $\left\{c_{1},\ldots ,c_{m}\right\}$. This variable is used only during the training stage and is the target to be inferred.

**Figure 2:**
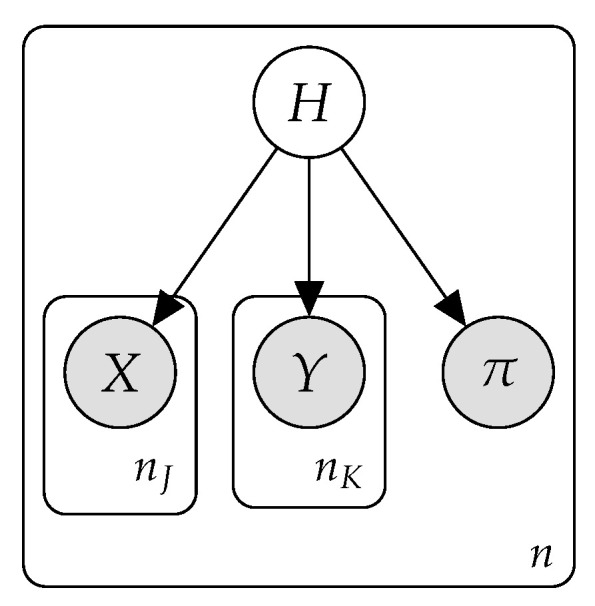
The proposed HNB model.

Figure 2 shows a plate-based representation of the proposed model with the different types of variables described above ($n=n_{J}+n_{K}$). The model assumes that each of these variable types follows a different conditional distribution given the root variable:

- Discrete variables follow a Multinomial distribution,
  $$P\left(X_{j}|H\right)⇝P\left(X_{j}|H=h_{z}\right)∼Mult\left({\left\{p\left(x_{j_{i}}^{h_{z}}\right)\right\}}_{i=1}^{r_{j}}\right),\;\;z=1,\ldots ,r_{H}\;$$
- Continuous variables follow a Normal or univariate Gaussian distribution,
  $$P\left(Y_{k}|H\right)⇝P\left(Y_{k}|H=h_{z}\right)∼N\left(\mu_{k}^{h_{z}},\sigma_{k}^{h_{z}}\right),\;\;z=1,\ldots ,r_{H}\;$$
- The ranking variable follows a Mallows distribution,
  $$P\left(\pi |H\right)⇝P\left(\pi |H=h_{z}\right)∼M\left(\pi_{0}^{h_{z}},\theta^{h_{z}}\right),\;\;z=1,\ldots ,r_{H}\;$$
- The hidden variable follows a Multinomial distribution,
  $$P\left(H\right)∼Mult({\left\{p\left(h_{z}\right)\right\}}_{z=1}^{r_{H}})$$

The parameters for each of the conditional distributions need to be estimated to perform inference using the model.

### 3.2. Parameter Estimation

As is common in most machine learning papers, we assume *i.i.d.* data. Furthermore, we also assume *complete* data, i.e., without missing values, both in the predictive and in the ranking variables. If there are missing values in the training data, they must be imputed previously to learn the model. The ranking variable can be imputed as described in [2]. Thus, we only deal with a hidden variable, *H*, and base our approach on the use of the *Expectation-Maximization* (*EM*) algorithm to estimate jointly the parameters of both the observed and hidden variables.

The EM algorithm [37] consists of two steps: the *expectation* step (*E* step), where the values for the hidden variable are estimated, and the *maximization* step (*M* step), where the parameters for the conditional distributions are obtained. Below, we describe these steps:

- **E step**: Under the assumption that the parameters of the model $\{\mu_{k}^{h_{z}}$, $\sigma_{k}^{h_{z}}$, $p(x_{j_{i}}^{h_{z}})$, $\pi_{0}^{h_{z}}$, $\theta^{h_{z}}$, $p(h_{z})\}$$k=1,\ldots ,n_{K}$, $j=1,\ldots ,n_{J}$, $i=1,\ldots ,r_{j}$, $z=1,\ldots ,r_{H}$, are known, the probability of an example $e_{t}=\left(x_{1,t},\ldots ,x_{n_{J},t},y_{1,t},\ldots ,y_{n_{K},t},\pi_{t}\right)$ being in a mixture $h_{z}$ is
  (3) $$\begin{array}{cc} P(h_{z}|x_{1,t},\ldots ,x_{n_{J},t},y_{1,t},\ldots ,y_{n_{K},t},\pi_{t}) & =\frac{1}{C}\cdot P\left(h_{z}\right)\cdot P\left(x_{1,t},\ldots ,x_{n_{J},t},y_{1,t},\ldots ,y_{n_{K},t},\pi_{t}|h_{z}\right)\\ & =\frac{1}{C}\cdot P\left(h_{z}\right)\cdot P\left(\pi_{t}|h_{z}\right)\cdot \prod_{j=1}^{n_{J}}P\left(x_{j,t}|h_{z}\right)\cdot \prod_{k=1}^{n_{K}}P\left(y_{k,t}|h_{z}\right)\\ & =\frac{1}{C}p\left(h_{z}\right)\cdot \frac{e^{-\theta^{h_{z}}\cdot D\left(\pi_{t},\pi_{0}^{h_{z}}\right)}}{\Psi (\theta^{h_{z}})}\cdot \prod_{j=1}^{n_{J}}p\left(x_{j,t}^{h_{z}}\right)\cdot \prod_{k=1}^{n_{K}}\frac{1}{\sigma_{k}^{h_{z}}\sqrt{2}}e^{-\frac{1}{2}{\left(\frac{y_{k,t}-\mu_{k}^{h_{z}}}{\sigma }\right)}^{2}} \end{array}$$
  Here, $C=\sum_{z=1}^{r_{H}}P\left(h_{z}\right)\cdot P\left(x_{1,t},\ldots ,x_{n_{J},t},y_{1,t},\ldots ,y_{n_{K},t},\pi_{t}|h_{z}\right)$ is the normalization constant.
- **M step**: Under the assumption that the probabilities of belonging to each mixture for all examples are known, the parameters of the model can be estimated as follows:
  - Multinomial parameters for the discrete variables. Each multinomial parameter $p(x_{j_{i}}^{h_{z}})$ is estimated by using MLE, where the count for each instance is weighted by the probability of $H=h_{z}$ given the instance.
  - Gaussian distribution parameters for the continuous variables. The parameters $\mu_{k}^{h_{z}}$ and $\sigma_{k}^{h_{z}}$ of the Gaussian distribution are estimated through MLE for each H=hz, weighting each instance by the probability of it being in the mixture.
  - Mallows distribution parameters for the ranking variable. For each component of the mixture $h_{z}$ of *H*, a Mallows distribution $M(\pi_{0}^{h_{z}},\theta^{h_{z}})$ must be estimated (see Section 2.3). In particular, $\pi_{0}^{h_{z}}$ is computed by applying a weighted version of the Borda count algorithm (points assigned to items are weighted by the probability of $H=h_{z}$ given the instance), and $\theta^{h_{z}}$ is calculated by using the Newton–Raphson numerical optimization method.
  - The mixture model probabilities P(H) are computed according to the weights $P(h_{z}|e_{t})$ for each mixture $h_{z}$ of *H* (see Equation (3)) by means of MLE.

**Stopping condition**: The EM algorithm can easily accommodate different types of stop conditions, most of them based on checking the convergence of some score (logarithm likelihood, accuracy, etc.).

### 3.3. Learning Process

In addition to the graphical structure and the parameter estimation already described, we also need to determine some kind of *structural* learning in order to find the inner structure of *H*, that is, its cardinality or number of states.

Basically, we follow a greedy technique by initializing $r_{H}$ to a certain number and then running consecutive executions of the EM algorithm with an increasing number of mixtures.

There are several points to discuss regarding the learning process: the initial value for $r_{H}$, the value used to increment $r_{H}$ between two consecutive iterations, the way the components of the mixture are initialized, and how the goodness of the model is evaluated and the final value of $r_{H}$ is selected. Below, we describe the two proposed schemes.

#### 3.3.1. Method A: HNBE-LR

First, we describe the scheme proposed in the preliminary (conference) version of this study [23], based on the learning process used in [36] and which basically wraps the EM method for parameter estimation. In Algorithm 1, we show our adaptation from the NB estimation algorithm [31] to the LR problem. The main characteristics of this method are as follows:
**Algorithm 1:** Method A: HNBE-LR
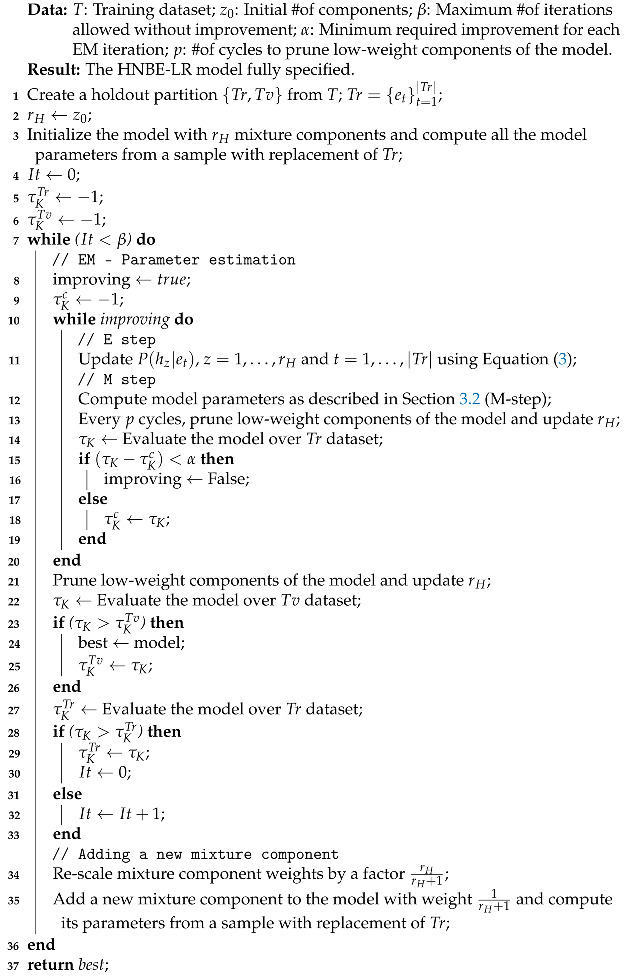

- It is a wrapper method. Thus, the data received is divided into training Tr and validation Tv datasets, using the *Kendall coefficient* $\tau_{K}$ to assess the models and parameterizations explored during the search.
- The search for the number of components of the mixture is carried out greedily. We start with an initial number of components $z_{0}$ and a new component is added at each iteration. However, low probability components are pruned both during the search and during the EM-based parameter estimation. The search stops when the obtained model does not improve the best one in a given number of consecutive iterations. The improvement is assessed by evaluating the current parameterized model over the training dataset Tr.
- Every time a new component is added to the mixture, the model parameters corresponding to this component are initialized from a set of instances of Tr obtained by using sampling with replacement.
- For each number of components in the mixture $r_{H}$ tried, an EM is run for parameter estimation. After each iteration (E and M steps), the model is evaluated using the Kendall coefficient $\tau_{K}^{Tr}$ for the training data Tr. A threshold on the difference between this value and the previous one is used to check convergence.
- When the number of components changes (either because of pruning low probability ones or because of the addition of a new one), the component weights are properly rescaled.

#### 3.3.2. Method B: HNB-LR

The results obtained in [23] shed light on certain drawbacks. The main one is that the algorithm reaches the stopping condition too soon, which results in a small number of components for the mixture. As the authors in [36] noted, in contrast to clustering (e.g., AutoClass [31]), a high number of mixture components is required to obtain an accurate approximation of the joint probability estimation.

Bearing this in mind, and also that the number of different values tried for $r_{H}$ must be small for reasons of efficiency, we propose an alternative scheme that we call *Method B*. As in Method A, the main idea is to wrap the EM algorithm by a search procedure to look for the number of components to be included in the mixture. In order to do that, we introduce important design modifications. Algorithms 2 and 3 show the scheme of this approach. Below, we comment on their main characteristics and differences with respect to Method A.

- In Method B, low probability components are not pruned, and so the EM algorithm is carried out in the search process (see Algorithm 2). Furthermore, the convergence of the EM algorithm is checked by using the log-likelihood (LL) of the data (Tr) given the model, that is, no wrapper evaluation is carried out to compute $\tau_{K}$ inside EM.
- The search process works in a wrapper style. Thus, we divide the data received into a training Tr and validation Tv datasets, and use $\tau_{K}$ to assess the models explored during the search.
- The search for the number of components is carried out greedily, but we now split it into two phases. The first is a forward search, where we evaluate the model with $r_{H}=2^{1},2^{2},2^{4},\ldots ,2^{10}$. We then select the best value $r_{H}^{\prime }$ according to $\tau_{K}^{Tv}$ and run a binary search between $\frac{r_{H}^{\prime }}{2}$ and $r_{H}^{\prime }$. Finally, the best value $r_{H}^{*}$ found in the binary search (see Section 5.2) according to $\tau_{K}^{Tv}$ is returned as the number of components for our model.
  The intuition behind this greedy search is to be efficient (at most, 20 values are tested) and to quickly try large values for $r_{H}$, as we identified this point as a shortcoming of Method A.
- Each time a new value for $r_{H}$ is tried, the process starts from scratch, that is, all the components are initialized simultaneously, instead of being added to the model as in Algorithm 1. To initialize the component parameters (probabilities and weights), *k*-means clustering processes [38] with $k=r_{H}$ are run, and the better one according to the minimal sum of distances between points and clusters centroids is selected. The instances associated with each cluster are used to initialize the corresponding mixture component.

**Algorithm 2:** Method: EM
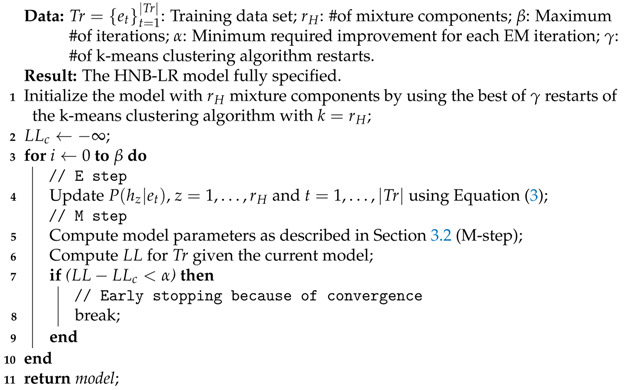

**Algorithm 3:** Method B: HNB-LR
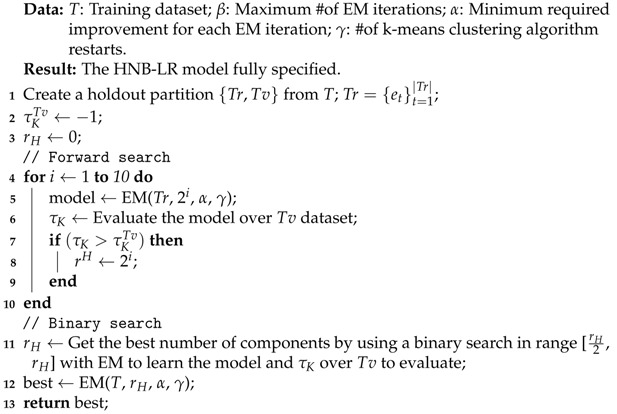


### 3.4. Inference Process

In the inference process, the method needs to predict the ranking associated with a given instance $e_{t}$. In our proposal, the probability of a ranking $\pi_{s}$ given $e_{t}$ can be obtained by marginalizing out variables until we obtain an expression for the posterior probability

$$P\left(\pi_{s}|e_{t}\right)\propto \sum_{z=1}^{r_{H}}\left(P\left(h_{z}\right)\cdot P\left(\pi_{s}|h_{z}\right)\cdot \prod_{j=1}^{n_{J}}P\left(x_{j,t}|h_{z}\right)\cdot \prod_{k=1}^{n_{K}}P\left(y_{k,t}|h_{z}\right)\right)$$

The outcome can then be obtained by using the MAP principle, that is, choosing the ranking $\pi^{*}$ which maximizes the score

$$\pi^{*}=\underset{\pi_{s}\in S_{m}}{argmax}\;P\left(\pi_{s}|e_{t}\right).$$

However, due to the possible high cardinality of $S_{m}$, we propose an approximate method:

1. Compute the probability a posteriori of each component of the mixture given the instance $e_{t}$:
  (4) $$P\left(h_{z}|e_{t}\right)\propto \prod_{j=1}^{n_{J}}P\left(x_{j,t}|h_{z}\right)\cdot \prod_{k=1}^{n_{K}}P\left(y_{k,t}|h_{z}\right)$$
2. Solve a generalized aggregation problem by using the weighted Borda count over the set of weighted rankings $\left\{\left(\pi_{0}^{h_{1}},w_{1}\right),\ldots ,\left(\pi_{0}^{h_{r_{H}}},w_{r_{H}}\right)\right\}$, that is, the consensus rankings associated with the components of the mixture, and taking as weight $w_{z}$, the probability a posteriori computed for the mixture component $P(h_{z}|e_{t})$.

## 4. Gaussian Mixture-Based Semi-Naive Bayes for Label Ranking

In this section, we go one step further by allowing interactions between the predictive variables. However, in order to maintain the complexity of the learning process under control, we decided to use a model in which, apart from identifying the number of mixture components, no structural learning is needed. Our proposal falls in the so-called *Semi-Naive Bayes* approach [30,39], and we restrict our study to continuous predictive variables. This constraint is quite natural in the LR problem, as all the benchmark datasets contain only continuous variables. In the future, we plan to adapt our method to also allow discrete predictive attributes, which, in general, means learning constrained graphical structures by limiting the number of dependencies allowed [30] or even dealing with *hybrid Bayesian networks* [40]. Learning PGMs with hidden variables is not an easy task, but there are several approaches in the literature, most based on the use of the *Structural EM* (*SEM*) algorithm [41].

### 4.1. Model Definition

Once we limit our model to contain only continuous predictive attributes $Y_{1},\ldots ,Y_{n_{K}}$, π, and *H*, and also avoid structural learning apart from $n_{H}$, we have to deal with representing the interactions between the variables. We maintain the interaction between π and the predictive variables to be channeled through the hidden variable *H*. Thus, explicit interactions are only allowed between the continuous attributes. As no structural learning of these relations is desired, we decided to model them by using a *multivariate Gaussian distribution*, $\mathrm{MN}(\vec{\mu },\Sigma )$. This has the advantage of having to estimate only ${n_{K}}^{2}+n_{K}$ parameters, as $n_{K}$ are the values in the vector of means $\vec{\mu }$ and ${n_{K}}^{2}$ in the covariance matrix.

In the literature, a *Gaussian Mixture Model* (*GMM*) [42] is a parametric probability density function represented as a weighted sum of Gaussian component densities, where each component density is a multivariate Gaussian function. Therefore, we take advantage of the widely used GMM to plug them into our PGM to deal with the LR problem. In the literature, we can find several variants of the GMM, which differ in the way the covariance matrix is constrained or not constrained. In particular, the standard or *full* GMM estimates a covariance matrix for each component with no additional constraint. On the other hand, in the *diag* variant, such a covariance matrix is constrained to be diagonal, which is equivalent to the NB assumption. The third option is to use a *tied* covariance matrix, which means estimating an unconstrained covariance matrix, but using it for all the components.

**Figure 3:**
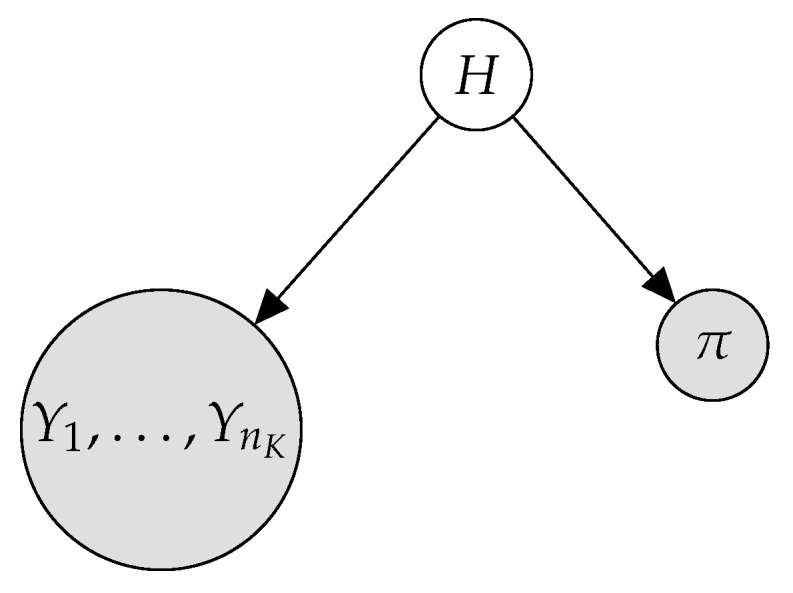
The proposed GMSNB model.

Figure 3 shows the graphical representation of the proposed *semi-naive Bayes* (*SNB*) model, where the large node including all the continuous attributes emphasizes the idea of modeling them jointly. The difference regarding the HNB presented in Section 3 is that the continuous variables now follow a multivariate Gaussian distribution given the root variable

$$P\left(Y|H\right)⇝P\left(Y|H=h_{z}\right)∼\mathrm{MN}\left({\vec{\mu }}^{h_{z}},\Sigma^{h_{z}}\right),\;\;z=1,\ldots ,r_{H}$$

where *Y* is the set of continuous variables $\left\{Y_{1},\ldots ,Y_{n_{K}}\right\}$, $\vec{\mu }$ is the vector of means for $Y_{1},\ldots ,Y_{n_{K}}$, and Σ is the $n_{K}\times n_{K}$ covariance matrix.

### 4.2. Parameter Estimation

As in the case of the proposed HNB algorithm, we use the EM algorithm to estimate the model parameters. Next, we point out the differences between this method and the univariate case (see Section 3.2).

- **E step**: Under the assumption that the parameters of the model $\{{\vec{\mu }}^{h_{z}}$, $\Sigma^{h_{z}}$, $\pi_{0}^{h_{z}}$, $\theta^{h_{z}}$, $p(h_{z})\}$, $z=1,\ldots ,r_{H}$, are known, the probability of an example $e_{t}=\left(y_{1,t},\ldots ,y_{n_{K},t},\pi_{t}\right)=\left({\vec{y}}_{t},\pi_{t}\right)$ being in a mixture $h_{z}$ is
  (5) $$\begin{array}{cc} P(h_{z}|{\vec{y}}_{t},\pi_{t}) & =\frac{1}{C}\cdot P\left(h_{z}\right)\cdot P\left({\vec{y}}_{t},\pi_{t}|h_{z}\right)\\ \; & =\frac{1}{C}\cdot P\left(h_{z}\right)\cdot P\left(\pi_{t}|h_{z}\right)\cdot P\left({\vec{y}}_{t}|h_{z}\right)\\ \; & =\frac{1}{C}\cdot P\left(h_{z}\right)\cdot M\left(\pi_{t}:\pi_{0}^{h_{z}},\theta^{h_{z}}\right)\cdot \mathrm{MN}\left({\vec{y}}_{t}:{\vec{\mu }}^{h_{z}},\Sigma^{h_{z}}\right) \end{array}$$
  where $C=\sum_{z=1}^{r_{H}}P\left(h_{z}\right)P\left({\vec{y}}_{t},\pi_{t}|h_{z}\right)$ is the normalization constant and $\mathrm{MN}({\vec{y}}_{t}:{\vec{\mu }}^{h_{z}},\Sigma^{h_{z}})$ stands for the probability density function of the multinormal distribution with parameters ${\vec{\mu }}^{h_{z}}$ and $\Sigma^{h_{z}}$ given by
  $$\mathrm{MN}\left(\vec{y}:{\vec{\mu }}^{h_{z}},\Sigma^{h_{z}}\right)=\frac{1}{\sqrt{{\left(2\right)}^{n_{K}}{\left|\Sigma \right|}^{h_{z}}}}e^{-\frac{1}{2}{\left(\vec{y}-{\vec{\mu }}^{h_{z}}\right)}^{T}{\left(\Sigma^{h_{z}}\right)}^{-1}\left(\vec{y}-{\vec{\mu }}^{h_{z}}\right)}$$
  Here, $\vec{y}$ is a configuration of values for variables $\left(Y_{1},\ldots ,Y_{n_{K}}\right)$, |Σ| is the determinant of Σ, and −1 and *T* denote the inverse and transpose matrix operators, respectively.
- **M step**: Under the assumption that the probabilities of belonging to each mixture for all the examples are known, the parameters of the model can be estimated as follows:
  - Continuous variables. Empirical means and covariance matrices are calculated in the standard way, using each instance being weighted by $w_{t}^{h_{z}}=P\left(h_{z}|{\vec{y}}_{t},\pi_{t}\right)$ according to the expressions
    $$N^{h_{z}}=\sum_{t=1}^{N}w_{t}^{h_{z}}$$
    $${\vec{\mu }}^{h_{z}}=\frac{1}{N^{h_{z}}}\sum_{t=1}^{N}w_{t}^{h_{z}}\cdot {\vec{y}}_{t}$$
    $$\Sigma^{h_{z}}=\frac{1}{N^{h_{z}}}\sum_{t=1}^{N}w_{t}^{h_{z}}\cdot \left({\vec{y}}_{t}-{\vec{\mu }}^{h_{z}}\right)\times {\left({\vec{y}}_{t}-{\vec{\mu }}^{h_{z}}\right)}^{T}$$
    Here, × stands for the usual matrix product.
    In the *tied* case, where all the components share the same covariance matrix, Σ, it is estimated as [43] (p. 71):
    $$\Sigma =\frac{1}{N}\sum_{z=1}^{r_{H}}\sum_{t=1}^{N}w_{t}^{h_{z}}\cdot \left({\vec{y}}_{t}-{\vec{\mu }}^{h_{z}}\right)\times {\left({\vec{y}}_{t}-{\vec{\mu }}^{h_{z}}\right)}^{T}$$

### 4.3. Learning Process

Method B, described in Section 3.3, is used to estimate the number of components for the mixture *H*. To do so, Algorithm 2 is modified as follows:

- In the E step, Equation (5) is used instead of Equation (3).
- In the M step, the expressions in Section 4.2 are used instead of the respective ones in Section 3.2.

### 4.4. Inference Process

The same inference process is used as in the HNB model (see Section 3.4). The only difference is that we now compute the posterior probability of each component of the mixture given the instance $e_{t}$ by using the multivariate probability density function instead of Equation (4).

(6) $$P\left(h_{z}|e_{t}\right)=P\left(h_{z}|{\vec{y}}_{t}\right)\propto \frac{1}{\sqrt{{\left(2\right)}^{n_{K}}\left|\Sigma^{h_{z}}\right|}}e^{-\frac{1}{2}{\left(\vec{y}-{\vec{\mu }}^{h_{z}}\right)}^{T}{\left(\Sigma^{h_{z}}\right)}^{-1}\left(\vec{y}-{\vec{\mu }}^{h_{z}}\right)}$$

## 5. Experimental Evaluation

In this section, we assess the mixture-based algorithms proposed to solve the LR problem. Below, we detail the datasets used, the algorithms tested, the methodology adopted, and the results obtained.

### 5.1. Datasets

Table 1 shows the main characteristics of the 21 datasets widely used as benchmark for the LR problem. The first 16 datasets were turned from multi-class (Type A) and regression (Type B) problems into the LR problem [2], while the last 5 datasets (Type R) correspond to real-world biological problems [10]. The columns #rankings and max #rankings represent the actual number of different rankings in the dataset and the maximum number of different rankings according to the number of classes (#classes), respectively. In the 21 datasets considered, all the predictive attributes (features) are continuous variables. A more detailed description of the datasets is provided at: https://scikit-lr.readthedocs.io/en/latest/user_guide/datasets.html#datasets (accessed on 29 March 2021).

### 5.2. Algorithms

In this study, we considered the following algorithms:

- The IBLR algorithm introduced in [2] (see Section 2.5). To identify the nearest neighbors, the Euclidean distance was used. To compute the prediction, the permutations associated with the *k*-nearest neighbors were weighted according to the neighbor’s (inverse) distance to the input instance. Although the IBLR algorithm belongs to the *lazy* paradigm of machine learning, we carried out model learning to select the number *k* of nearest neighbors. We applied the following process using a fivefold cross-validation method (5-cv) over the training dataset to assess the goodness of each candidate value:
  1. We started with k=5 and doubled it while the score was improving. From this process, we obtained $k_{l}$ and $k_{r}$, that is, the number of nearest neighbors leading to the best score (the penultimate value tested) and the one stopping the iterative process (the last value tested), respectively.
  2. We applied a binary search in the range [$k_{l}$, $k_{r}$]. In this process, we took $k_{m}=\lfloor \frac{k_{l}+k_{r}}{2}\rfloor$, and if the score improved for $k_{m}$ with respect to $k_{l}$, we then repeated this recursive process using the range $\left[k_{m},k_{r}\right]$. Otherwise, the range $\left[k_{l},k_{m}\right]$ was used.
  We kept the number of nearest neighbors that led to the best score.
- The LRT algorithm introduced in [2] (see Section 2.6).
- The HNBE-LR algorithm introduced in [23] (see Section 3.3.1). Note that all the attributes of the datasets are continuous variables. Thus, the parameters of the model were estimated by means of Gaussian distribution parameters (HNBE-LR-G). The hyperparameter values were $z_{0}=5$, β=1, α=0.001, p=5. The holdout for the training and validation datasets was 75%/25%.
- The HNB-LR algorithm (see Section 3.3.2). As in the previous case, we estimated the conditional probability distributions with Gaussian univariate distributions (HNB-LR-G). Furthermore, we binned the continuous variables using equal-frequency (HNB-LR-F), equal-width (HNB-LR-W), and entropy-based [44] (HNB-LR-E) discretization techniques, estimating the parameters of the model with Multinomial distribution parameters. The number of bins was set to 5 for equal-width and equal-frequency cases. The holdout for the training and validation datasets was 80%/20%. The γ value was fixed to 10.
- The GMSNB-LR algorithm (see Section 4), with a different covariance matrix for each component, *full* approach (GMSNB-LR-F), and sharing the same covariance matrix between all the components of the mixture, *tied* approach (GMSNB-LR-T). The holdout for the training and validation datasets was 80%/20%. The γ value was fixed to 10.

### 5.3. Methodology

We adopted the following design decisions:

- We used five repetitions of a tenfold cross-validation method (5 × 10-cv) to assess the algorithms.
- We used the Kendall rank correlation coefficient $\tau_{K}$ as goodness score: the higher, the better (see Section 2.2).
- To properly analyze the results, we carried out the standard statistical analysis procedure for machine learning [45,46], using the exreport tool [47]. The procedure is divided into two steps:
  1. First, we carried out a *Friedman test* [48] with significance level α=0.05. If the obtained *p*-value ≤α, then we rejected the null hypothesis $H_{0}$ and concluded that at least one algorithm is not equivalent to the rest.
  2. Second, once the previous step rejected $H_{0}$, we applied a post hoc test using the *Holm’s procedure* [49] to discover the outstanding algorithms. This test compares all the algorithms against the control algorithm, that is, the one ranked first by the Friedman test.
- We executed the experiments on computers running the CentOS Linux 7 operating system with an Intel(R) Xeon(R) E5–2630 CPU running at 2.40 GHz, and with 16 GB of RAM memory.

### 5.4. Results

In this section, we present and analyze the results obtained. We focus on accuracy ($\tau_{K}$ score) and CPU time.

#### 5.4.1. Accuracy

First, we analyze the results obtained by the HNB-LR algorithms. The $\tau_{K}$ accuracy results for this family of algorithms are shown in Table 2. The cells contain the average and standard deviation over the test sets of the cross validation method for the rank correlation coefficient $\tau_{K}$ between the real and predicted permutations. The boldfaced values correspond to the algorithm(s) achieving the best mean accuracy for each dataset.

We base our analysis on the statistical procedure described in Section 5.3:

1. The *p*-value obtained in the Friedman test was 3.613 ×$10^{-5}$). Therefore, the null hypothesis ($H_{0}$) was rejected, and at least one of the tested algorithms was different.
2. Table 3 shows the results for the post hoc test by taking HNB-LR-G, the algorithm ranked first by the Friedman test, as the control. The columns *rank* and *p*-value represent the ranking obtained by the Friedman test and the *p*-value adjusted by Holm’s procedure, respectively. The columns *win*, *tie*, and *loss* contain the number of times that the control algorithm wins, ties, and loses with respect to the row-wise algorithm. The *p*-values for the non-rejected null hypothesis are boldfaced.

According to these results and the statistical analysis performed, we can conclude the following.

- The HNBE-LR-G algorithm is the worst method. The reason is obvious if we analyze Table 4, where we show the number of components (on average) selected for each algorithm. It is clear that this number is too small for HNBE-LR-G, which clearly suffers from premature early stopping. Furthermore, we must recall that this algorithm is the only one which prunes low weight components during its performance. As a consequence, HNBE-LR-G does not obtain a good probability estimation.
- The HNB-LR-G algorithm is ranked first and is statistically different to the HNB-LR-W and HNBE-G algorithms. In the case of HNB-LR-W, the reason is not the number of selected components, but the equal-width discretization carried out, which produces poor binning in comparison, for example, with the supervised entropy-based method.
- Although the HNB-LR-G algorithm is ranked first, the post hoc test reveals no significant difference with respect to the HNB-LR-E and HNB-LR-F algorithms. This opens the door to future research on more complex Bayesian network structures.
- As stated in [36], a large number of components are required to obtain a good probability estimation and, in our problem, a good ranking prediction.

Once we have determined that the best HNB-LR algorithms are HNB-LR-G, HNB-LR-E, and HNB-LR-F, we introduce the two algorithms allowing interactions among the predictive attributes in the study, that is, those based on the use of the multivariate Gaussian distribution to jointly model the (numerical) attributes: GMSNB-LR-F and GMSNB-LR-T. The results are shown in the two leftmost columns of Table 5. To complete the comparison, we also show the results for the two state-of-the-art LR classifiers [2] described in Section 2.5 and Section 2.6.

Again, we applied the statistical analysis procedure described in Section 5.3, including also the three HNB-LR algorithms selected from the previous study:

1. The *p*-value obtained for the Friedman test was 2.503 × $10^{-7}$. Therefore, we rejected the null hypothesis ($H_{0}$), i.e., at least one algorithm is different to the rest.
2. As IBLR is ranked first by the Friedman test, we took it as the control and performed a post hoc test using Holm’s procedure. Table 6 shows the results for the post hoc test.

Considering these results, we can conclude the following.

- The IBLR algorithm is ranked first, being statistically different to all the tested algorithms except GMSNB-LR-T. Note that IBLR is a fine-tuned algorithm, as can be observed from the number of neighbors selected for each dataset (see Table 4), which are far from standard values (3,5,…).
- The GMSNB-LR-T algorithm has a remarkable performance, being non-significantly different to IBLR. This is a very important finding because, as recognized in the literature, the instance-based algorithm generally outperforms the model-based algorithms, being necessary to use ensembles of the LRT algorithm to compete with it [14].
- The GMSNB-LR-T algorithm also has in its favor that it is able to cope with all the tested datasets, while the experiments for IBLR cannot finish in a maximum of 168 h (one week) for fried dataset (notice the *empty* cell in Table 5). As can be observed, fried is the largest dataset in our experiments, which reveals the disadvantage of using IBLR for larger domains.
- The GMSNB-LR-T algorithm behaves better than GMSNB-LR-F, which is also ranked behind HNB-LR-G. Two explanations are plausible for this behavior: First, the amount of data considered is limited, so it can be scarce for the estimation of many covariance matrices when the number of components grows. Second, it is well known that increasing the number of components can be enough to model the correlations between the features. In fact, if we observe Table 4, we realize that the numbers of components selected for GMSNB-LR-T and HNB-LR-G are noticeably greater than that selected for GMSNB-LR-F.

#### 5.4.2. Time

In this study, we consider model-based and instance-based machine learning algorithms, which clearly distribute the CPU time for the learning and inference steps differently. Although the CPU time for the whole process (learning from the training dataset and validating with test dataset) is generally reported, we separate the CPU time for the learning and inference steps because (i) a model is learnt once but queried many times and (ii) most real-world applications require online predictions but allow for offline fitting. Table 7 and Table 8 show the average CPU time for the learning and inference steps. In light of these results, we can conclude the following.

- The HNBE-LR-G algorithm is the fastest method during the learning step because it suffers from premature early stopping, which gives rise to a fast but poor algorithm. On the other hand, the LRT algorithm is the fastest method during the inference step, which is a common situation for tree-based algorithms.
- The IBLR algorithm is faster than the HNB-LR and GMSNB-LR algorithms in the learning step. However, during inference, the IBLR algorithm computes the distance between the input instance and the instances in the training dataset, which clearly increases the CPU time required by the algorithm.
- The GMSNB-LR-F algorithm is generally faster than the GMSNB-LR-T algorithm, both in learning and inference. This is due to the number of components selected by the GMSNB-LR-F algorithm in comparison to the GMSNB-LR-T algorithm. In a similar way, the HNB-LR-G algorithm is faster than the HNB-LR-F, HNB-LR-W, and HNB-LR-E algorithms, as the the latter ones apply a discretization procedure prior to the learning and inference steps.

## 6. Limitations

As observed in the previous section, allowing interactions between the predictive variables represents a crucial issue with respect to the univariate case. Actually, our mixture-based model emerges as competitive with IBLR. However, there are still some weaknesses in our study/proposal:

- The CPU time data shown in Table 7 suggest that our method does not scale as would be desirable. In fact, it seems that the number of instances has a greater influence on the CPU time than the number of variables. However, more analysis is needed to clarify this point. Feature subset selection and stratification could lead to scalability improvements.
- Interactions between the predictive variables are limited to the numerical ones. Allowing interactions among the discrete variables and also mixed interactions should be studied. The literature on Bayesian network classifiers [30,39] and hybrid Bayesian networks may help in this task [50].
- Currently, the method only works with *complete rankings*. Nevertheless, in real-world applications it is usual to allow the agent to rank only certain labels. We think our techniques can be adapted to deal with incomplete rankings.

## 7. Conclusions

This study explores the use of mixture-based algorithms to solve the LR problem. The main problem is to model the target variable, as it takes values in the set of permutations defined over the class variable. We solve this shortcoming by introducing a hidden variable as root, so all the variables can be modeled by using conditional distributions. In particular, we base our approach on the Naive Bayes structure, with the hidden variable being the root of the model. We then go a step further by allowing interactions between the (numerical) predictive variables, thus designing a Semi-Naive Bayes model. Learning algorithms based on the well-known EM estimation principle are proposed for both cases. The inference is designed as a combination of probabilistic inference and rank aggregation.

From the experimental evaluation, we observe that the Naive Bayes approach is comparable in score to the decision trees for the LR problem, while the Semi-Naive Bayes approach (in particular, the one sharing a single covariance matrix among all the components) outperforms the Naive Bayes and decision tree-based algorithms, being also competitive with the state-of-the-art model-free algorithm based on the nearest neighbors method (IBLR). The good performance of this algorithm (GMSNB-LR-T) with respect to IBLR is reinforced by its better behavior at the inference stage, where IBLR needs a great amount of time when facing large datasets.

As future research, we propose two possible extensions to this work: First, we plan to extend our approach to cope with incomplete data in the ranking variable, that is, cases in which not all the labels are ranked in the instances of the training set. In the literature, this step is solved by *completing* those rankings before learning the model. However, we think this step can be introduced in the EM algorithm. In fact, model-based algorithms have shown better behavior than model-free ones when learning from incomplete rankings, so open the door to future promising research. Second, we plan to extend the mixture-based algorithms to the LR problems whose target ranking is a partial or bucket order, that is, a ranking in which some labels can be tied. This problem, which is generally termed the *Partial Label Ranking* (*PLR*) problem [51,52], introduces new challenges, such as the infeasibility of using the Mallows distribution to model the target variable.

## Figures and Tables

**Table 1:** Description of the datasets.

Dataset	Type	#Instances	#Features	#Classes	#Rankings	Max #Rankings
authorship	A	841	70	4	17	4!
bodyfat	B	252	7	7	236	7!
calhousing	B	20,640	4	4	24	4!
cpu	B	8192	6	5	119	5!
elevators	B	16,599	9	9	131	9!
fried	B	40,769	9	5	120	5!
glass	A	214	9	6	30	6!
housing	B	506	6	6	112	6!
iris	A	150	4	3	5	3!
pendigits	A	10,992	16	10	2081	10!
segment	A	2310	18	7	135	7!
stock	B	950	5	5	51	5!
vehicle	A	846	18	4	18	4!
vowel	A	528	10	11	294	11!
wine	A	178	13	3	5	3!
wisconsin	B	194	16	16	194	16!
spo	R	2465	24	11	2361	11!
heat	R	2465	24	6	622	6!
dtt	R	2465	24	4	24	4!
cold	R	2465	24	4	24	4!
diau	R	2465	24	7	967	7!

**Table 2:** Mean accuracy for each HNB-LR algorithm.

Dataset	HNBE-LR-G	HNB-LR-G	HNB-LR-F	HNB-LR-W	HNB-LR-E
authorship	0.907 (±0.028)	**0.919 (±0.018)**	0.905 (±0.021)	0.905 (±0.019)	0.909 (±0.017)
bodyfat	0.078 (±0.074)	**0.128 (±0.063)**	0.115 (±0.066)	0.116 (±0.062)	0.117 (±0.068)
calhousing	0.171 (±0.018)	0.303 (±0.024)	0.278 (±0.009)	0.198 (±0.011)	**0.343 (±0.013)**
cpu	0.360 (±0.023)	0.435 (±0.013)	**0.461 (±0.013)**	0.334 (±0.016)	0.459 (±0.013)
elevators	0.646 (±0.025)	**0.728 (±0.014)**	0.695 (±0.011)	0.664 (±0.013)	0.688 (±0.016)
fried	0.489 (±0.105)	**0.895 (±0.034)**	0.367 (±0.308)	0.404 (±0.303)	0.812 (±0.014)
glass	0.788 (±0.067)	0.793 (±0.061)	**0.849 (±0.051)**	0.834 (±0.057)	0.846 (±0.048)
housing	0.400 (±0.116)	**0.734 (±0.034)**	0.667 (±0.045)	0.639 (±0.041)	0.711 (±0.044)
iris	**0.963 (±0.060)**	0.962 (±0.048)	0.845 (±0.082)	0.900 (±0.056)	0.866 (±0.086)
pendigits	0.721 (±0.026)	0.914 (±0.003)	0.916 (±0.002)	0.912 (±0.003)	**0.920 (±0.003)**
segment	0.656 (±0.096)	0.926 (±0.008)	0.909 (±0.009)	0.928 (±0.008)	**0.939 (±0.007)**
stock	0.791 (±0.040)	**0.910 (±0.016)**	0.852 (±0.022)	0.861 (±0.016)	0.885 (±0.016)
vehicle	0.744 (±0.054)	0.790 (±0.038)	0.806 (±0.037)	0.798 (±0.059)	**0.810 (±0.033)**
vowel	0.545 (±0.067)	0.817 (±0.046)	**0.865 (±0.027)**	0.863 (±0.028)	0.616 (±0.092)
wine	**0.935 (±0.044)**	0.935 (±0.054)	0.927 (±0.064)	0.915 (±0.076)	0.928 (±0.053)
wisconsin	0.295 (±0.070)	0.355 (±0.050)	**0.373 (±0.046)**	0.334 (±0.055)	0.324 (±0.093)
cold	0.071 (±0.039)	**0.080 (±0.035)**	0.080 (±0.037)	0.073 (±0.037)	0.076 (±0.030)
diau	0.215 (±0.023)	**0.219 (±0.022)**	0.215 (±0.025)	0.219 (±0.024)	0.217 (±0.023)
dtt	0.119 (±0.034)	**0.120 (±0.034)**	0.114 (±0.033)	0.107 (±0.030)	0.119 (±0.031)
heat	0.054 (±0.025)	**0.061 (±0.028)**	0.057 (±0.026)	0.055 (±0.027)	0.059 (±0.024)
spo	0.147 (±0.016)	0.146 (±0.015)	**0.148 (±0.016)**	0.146 (±0.015)	0.148 (±0.015)

**Table 3:** Results of the post hoc test for the mean accuracy of HNB-LR algorithms.

Method	Rank	*p*-Value	Win	Tie	Loss
HNB-LR-G	2.05	-	-	-	-
HNB-LR-E	2.33	**5.5818** × $10^{-1}$	14	0	7
HNB-LR-F	2.86	**1.9422** × $10^{-1}$	14	0	7
HNB-LR-W	3.62	3.8394 × $10^{-3}$	16	0	5
HNBE-LR-G	4.14	7.0206 × $10^{-5}$	18	0	3

**Table 4:** Mean number of components for each mixture-based algorithm and mean number of nearest neighbors for the IBLR algorithm.

Dataset	HNBE-LR-G	HNB-LR-G	HNB-LR-F	HNB-LR-W	HNB-LR-E	GMSNB-LR-F	GMSNB-LR-T	IBLR
authorship	5.82 (±0.87)	28.66 (±24.98)	25.08 (±21.39)	22.18 (±21.95)	38.60 (±45.37)	3.26 (±0.44)	44.92 (±49.51)	6.52 (±1.62)
bodyfat	5.88 (±0.90)	36.74 (±27.42)	21.04 (±17.29)	24.92 (±23.04)	20.14 (±32.20)	20.66 (±14.57)	42.20 (±30.10)	28.68 (±13.49)
calhousing	7.12 (±1.67)	280.78 (±103.40)	357.72 (±84.48)	119.56 (±59.87)	461.96 (±81.83)	257.90 (±116.43)	453.58 (±88.94)	27.24 (±6.85)
cpu	6.60 (±1.84)	142.62 (±106.17)	153.10 (±83.49)	17.40 (±12.02)	230.06 (±116.99)	60.94 (±45.28)	372.62 (±129.13)	46.52 (±8.61)
elevators	5.44 (±0.67)	216.10 (±78.63)	95.30 (±42.99)	105.98 (±45.74)	134.12 (±49.31)	52.12 (±18.61)	219.50 (±75.32)	27.10 (±5.43)
fried	7.14 (±2.19)	500.82 (±36.01)	167.34 (±138.34)	171.58 (±131.69)	408.98 (±105.35)	257.62 (±21.08)	475.22 (±92.00)	
glass	5.34 (±0.56)	14.72 (±11.81)	74.56 (±37.55)	42.40 (±17.82)	26.14 (±10.63)	5.84 (±5.63)	48.32 (±13.45)	5.26 (±0.53)
housing	5.56 (±0.67)	64.08 (±19.45)	79.46 (±47.78)	68.52 (±47.02)	38.76 (±36.03)	45.94 (±25.61)	116.60 (±28.84)	39.88 (±6.94)
iris	5.78 (±0.71)	14.44 (±9.45)	21.46 (±14.90)	17.12 (±10.96)	16.36 (±12.78)	8.50 (±2.39)	21.62 (±16.59)	8.46 (±2.00)
pendigits	6.22 (±1.31)	503.20 (±29.99)	484.00 (±55.38)	451.62 (±87.11)	484.00 (±49.35)	126.16 (±6.82)	509.44 (±18.10)	6.18 (±0.56)
segment	5.56 (±0.61)	166.98 (±55.22)	341.02 (±138.26)	303.68 (±145.76)	383.14 (±139.14)	45.10 (±16.55)	371.08 (±109.12)	8.16 (±1.74)
stock	6.16 (±1.54)	123.42 (±48.48)	99.66 (±33.97)	56.60 (±31.58)	95.54 (±30.49)	45.96 (±13.12)	244.78 (±107.07)	5.66 (±1.15)
vehicle	6.92 (±1.63)	48.06 (±48.45)	239.58 (±169.52)	179.26 (±171.77)	111.72 (±114.48)	10.34 (±4.96)	191.38 (±154.49)	8.80 (±2.22)
vowel	6.04 (±1.19)	96.70 (±27.75)	239.58 (±32.12)	231.36 (±38.00)	132.70 (±60.35)	13.28 (±4.44)	251.86 (±13.70)	5.98 (±0.98)
wine	5.48 (±0.65)	11.76 (±13.61)	12.22 (±12.34)	16.30 (±17.10)	11.40 (±13.93)	3.68 (±1.25)	9.58 (±12.81)	7.16 (±2.48)
wisconsin	5.88 (±1.24)	23.10 (±14.23)	21.10 (±13.76)	49.90 (±41.21)	45.38 (±27.82)	4.60 (±1.85)	35.84 (±15.83)	14.24 (±4.75)
cold	6.28 (±1.40)	84.84 (±107.13)	72.00 (±99.91)	84.70 (±109.52)	98.42 (±148.12)	83.00 (±143.73)	229.26 (±134.65)	23.44 (±22.88)
diau	5.76 (±1.04)	13.24 (±20.64)	23.50 (±50.16)	15.92 (±23.00)	14.58 (±14.84)	18.40 (±15.89)	35.76 (±48.31)	132.04 (±40.12)
dtt	5.50 (±0.84)	104.22 (±154.80)	82.84 (±118.50)	120.76 (±148.76)	20.50 (±27.44)	36.52 (±46.53)	116.42 (±152.29)	101.80 (±27.78)
heat	6.24 (±1.15)	71.08 (±133.42)	74.44 (±116.93)	66.44 (±128.37)	77.72 (±127.87)	31.48 (±80.92)	79.68 (±135.62)	23.14 (±13.40)
spo	5.90 (±0.95)	10.16 (±9.16)	9.24 (±12.53)	11.26 (±13.44)	12.62 (±15.67)	18.28 (±31.55)	6.96 (±5.76)	354.66 (±314.51)

**Table 5:** Mean accuracy for the GMSNB-LR, IBLR, and LRT algorithms.

Dataset	GMSNB-LR-F	GMSNB-LR-T	IBLR	LRT
authorship	0.838 (±0.033)	0.925 (±0.019)	**0.935 (±0.014)**	0.883 (±0.024)
bodyfat	0.080 (±0.078)	0.151 (±0.074)	**0.230 (±0.055)**	0.151 (±0.066)
calhousing	0.295 (±0.020)	0.284 (±0.017)	**0.351 (±0.010)**	0.319 (±0.012)
cpu	0.418 (±0.016)	0.432 (±0.027)	**0.506 (±0.013)**	0.404 (±0.014)
elevators	0.774 (±0.017)	**0.780 (±0.009)**	0.730 (±0.006)	0.668 (±0.010)
fried	0.908 (±0.025)	**0.927 (±0.014)**		0.727 (±0.103)
glass	0.790 (±0.061)	0.879 (±0.059)	0.864 (±0.051)	**0.902 (±0.040)**
housing	0.695 (±0.044)	0.782 (±0.029)	0.716 (±0.031)	**0.811 (±0.029)**
iris	0.925 (±0.053)	0.962 (±0.035)	**0.963 (±0.042)**	0.953 (±0.044)
pendigits	0.891 (±0.003)	0.927 (±0.002)	**0.943 (±0.002)**	0.942 (±0.002)
segment	0.884 (±0.015)	0.947 (±0.008)	**0.961 (±0.005)**	0.955 (±0.006)
stock	0.894 (±0.015)	0.922 (±0.014)	**0.926 (±0.014)**	0.898 (±0.016)
vehicle	0.721 (±0.051)	0.802 (±0.042)	**0.860 (±0.026)**	0.818 (±0.040)
vowel	0.589 (±0.039)	**0.906 (±0.015)**	0.889 (±0.018)	0.753 (±0.033)
wine	0.937 (±0.054)	**0.944 (±0.042)**	0.941 (±0.048)	0.870 (±0.078)
wisconsin	0.244 (±0.085)	0.336 (±0.108)	**0.499 (±0.041)**	0.374 (±0.040)
cold	0.072 (±0.036)	**0.090 (±0.042)**	0.090 (±0.035)	0.048 (±0.031)
diau	0.222 (±0.025)	0.219 (±0.025)	**0.234 (±0.026)**	0.129 (±0.022)
dtt	0.124 (±0.030)	0.119 (±0.032)	**0.159 (±0.033)**	0.080 (±0.033)
heat	0.064 (±0.029)	0.046 (±0.025)	**0.070 (±0.022)**	0.039 (±0.023)
spo	0.148 (±0.016)	0.148 (±0.015)	**0.149 (±0.017)**	0.090 (±0.018)

**Table 6:** Results of the post hoc test for the mean accuracy of the algorithms.

Method	Rank	*p*-Value	Win	Tie	Loss
IBLR	1.76	-	-	-	-
GMSNB-LR-T	2.93	**8.012** × $10^{-2}$	14	0	7
HNB-LR-G	3.98	1.791 × $10^{-3}$	19	0	2
LRT	4.52	1.029 × $10^{-5}$	18	0	3
HNB-LR-E	4.67	5.271 × $10^{-5}$	20	0	1
GMSNB-LR-F	5.00	5.955 × $10^{-2}$	19	0	2
HNB-LR-F	5.14	2.369 × $10^{-6}$	20	0	1

**Table 7:** Mean CPU time (in seconds) for the learning step of each algorithm.

Dataset	HNBE-LR-G	HNB-LR-G	HNB-LR-F	HNB-LR-W	HNB-LR-E	GMSNB-LR-F	GMSNB-LR-T	IBLR	LRT
authorship	1.705 ±0.532	61.196 ±9.992	89.829 ±12.334	87.027 ±12.214	89.588 ±17.826	45.615 ±12.528	63.305 ±26.925	**0.750 ±0.123**	6.417 ±0.359
bodyfat	**0.242 ±0.091**	5.225 ±1.886	14.765 ±5.212	19.063 ±9.117	26.695 ±14.450	4.178 ±1.082	7.411 ±2.700	0.408 ±0.117	0.459 ±0.021
calhousing	**24.985 ±10.129**	4460.303 ±2556.481	934.879 ±403.026	549.931 ±330.861	924.278 ±193.472	2477.003 ±894.089	1640.548 ±879.352	222.602 ±18.316	617.978 ±95.233
cpu	**10.822 ±6.953**	832.624 ±453.921	434.776 ±115.788	284.175 ±155.825	334.790 ±92.549	880.115 ±271.609	1469.499 ±588.102	53.066 ±1.789	119.613 ±7.050
elevators	**15.681 ±4.200**	2252.878 ±909.937	3878.157 ±1111.013	2912.576 ±1199.323	1822.243 ±564.493	3696.911 ±817.021	1974.011 ±467.630	154.594 ±9.019	1685.996 ±174.829
fried	**51.613 ±29.227**	6783.099 ±4193.126	3050.954 ±1459.152	8534.450 ±3529.126	3255.716 ±1247.376	3836.842 ±625.103	7345.125 ±4261.981		6049.934 ±3015.594
glass	**0.139 ±0.037**	4.784 ±1.416	21.871 ±6.983	24.472 ±6.890	21.390 ±4.724	5.001 ±0.877	12.855 ±3.936	0.142 ±0.003	0.439 ±0.038
housing	**0.278 ±0.070**	33.743 ±9.953	74.223 ±23.715	77.063 ±36.041	72.623 ±24.394	12.118 ±4.511	37.575 ±20.451	1.011 ±0.129	0.843 ±0.035
iris	0.119 ±0.031	5.103 ±1.850	5.940 ±2.908	8.612 ±2.794	10.060 ±4.408	2.891 ±0.520	3.740 ±1.200	0.146 ±0.029	**0.024 ±0.015**
pendigits	**14.921 ±6.446**	4958.630 ±533.195	7526.738 ±1086.477	3363.739 ±1379.314	2181.268 ±503.646	1370.683 ±169.772	2171.361 ±377.718	37.522 ±0.314	137.337 ±3.893
segment	**1.893 ±0.633**	306.300 ±71.676	614.536 ±388.197	297.176 ±96.372	633.483 ±207.526	155.155 ±31.039	680.656 ±198.149	3.290 ±0.712	43.915 ±0.459
stock	1.153 ±0.642	78.335 ±18.392	206.016 ±20.207	203.993 ±29.806	215.489 ±23.562	51.965 ±10.650	110.541 ±46.287	**0.678 ±0.078**	1.767 ±0.044
vehicle	1.361 ±0.645	46.526 ±17.221	75.399 ±19.064	131.554 ±42.902	187.489 ±51.417	28.025 ±13.600	120.622 ±65.717	**0.784 ±0.162**	2.614 ±0.201
vowel	0.581 ±0.247	39.580 ±5.972	72.241 ±8.261	76.060 ±10.918	189.684 ±98.049	22.197 ±2.436	47.121 ±6.398	**0.364 ±0.004**	5.231 ±0.136
wine	**0.125 ±0.039**	1.565 ±0.502	4.887 ±1.482	5.235 ±2.170	6.360 ±2.088	1.264 ±0.129	1.614 ±0.468	0.132 ±0.029	0.250 ±0.095
wisconsin	**0.265 ±0.155**	17.549 ±3.963	13.207 ±2.893	20.881 ±10.524	40.325 ±9.262	6.196 ±0.661	13.596 ±4.252	0.287 ±0.060	2.414 ±0.081
cold	**3.400 ±1.779**	318.876 ±133.326	373.824 ±192.488	287.441 ±121.705	212.068 ±85.827	309.730 ±195.366	342.582 ±154.869	5.489 ±2.379	558.146 ±268.333
diau	**2.485 ±1.233**	234.570 ±60.736	296.337 ±85.944	175.379 ±46.762	273.500 ±88.171	266.421 ±62.646	176.723 ±60.193	14.260 ±2.636	509.939 ±173.209
dtt	**2.308 ±0.900**	320.371 ±164.964	440.020 ±233.137	317.019 ±167.063	155.423 ±32.987	246.605 ±80.833	228.628 ±119.854	11.159 ±1.455	436.914 ±52.761
heat	**3.444 ±1.448**	323.654 ±161.205	359.967 ±148.471	243.728 ±119.877	293.548 ±147.877	250.223 ±92.645	226.749 ±144.860	6.322 ±1.336	149.847 ±22.709
spo	**3.106 ±1.114**	345.584 ±57.367	364.797 ±97.080	238.538 ±47.706	357.678 ±121.603	338.189 ±90.514	218.729 ±34.441	33.453 ±19.871	330.353 ±61.667

**Table 8:** Mean CPU time (in seconds) for the inference step of each algorithm.

Dataset	HNBE-LR-G	HNB-LR-G	HNB-LR-F	HNB-LR-W	HNB-LR-E	GMSNB-LR-F	GMSNB-LR-T	IBLR	LRT
authorship	0.007 ±0.001	0.012 ±0.012	0.062 ±0.041	0.057 ±0.034	0.044 ±0.047	0.008 ±0.009	0.025 ±0.026	0.022 ±0.000	**0.002 ±0.000**
bodyfat	**0.001 ±0.000**	0.002 ±0.000	0.012 ±0.012	0.008 ±0.010	0.007 ±0.009	0.003 ±0.002	0.005 ±0.004	0.006 ±0.001	**0.001 ±0.000**
calhousing	**0.053 ±0.003**	0.409 ±0.217	0.682 ±0.197	0.376 ±0.177	0.617 ±0.154	0.348 ±0.162	0.354 ±0.122	3.675 ±0.033	0.116 ±0.011
cpu	**0.024 ±0.002**	0.069 ±0.038	0.219 ±0.121	0.068 ±0.025	0.263 ±0.121	0.067 ±0.031	0.260 ±0.094	0.713 ±0.020	0.033 ±0.002
elevators	**0.059 ±0.002**	0.152 ±0.049	0.321 ±0.117	0.252 ±0.098	0.128 ±0.040	0.214 ±0.055	0.345 ±0.102	2.620 ±0.027	0.243 ±0.088
fried	**0.124 ±0.013**	0.779 ±0.362	0.328 ±0.249	1.016 ±0.750	1.009 ±0.493	0.437 ±0.033	1.250 ±0.798		0.264 ±0.263
glass	**0.001 ±0.000**	0.002 ±0.003	0.021 ±0.013	0.015 ±0.013	0.008 ±0.008	0.003 ±0.004	0.009 ±0.008	0.004 ±0.000	**0.001 ±0.000**
housing	0.002 ±0.001	0.011 ±0.011	0.026 ±0.014	0.018 ±0.011	0.014 ±0.013	0.006 ±0.005	0.024 ±0.020	0.012 ±0.001	**0.001 ±0.000**
iris	**0.001 ±0.000**	0.004 ±0.007	0.005 ±0.006	0.005 ±0.006	0.003 ±0.006	0.003 ±0.004	0.004 ±0.004	0.003 ±0.000	**0.001 ±0.000**
pendigits	0.055 ±0.004	0.533 ±0.120	1.380 ±0.274	0.648 ±0.357	0.346 ±0.049	0.166 ±0.041	0.570 ±0.111	1.266 ±0.013	**0.025 ±0.000**
segment	0.011 ±0.002	0.049 ±0.019	0.093 ±0.046	0.135 ±0.080	0.191 ±0.079	0.045 ±0.016	0.190 ±0.062	0.085 ±0.002	**0.005 ±0.000**
stock	0.003 ±0.000	0.015 ±0.011	0.029 ±0.013	0.021 ±0.014	0.033 ±0.013	0.022 ±0.014	0.072 ±0.030	0.020 ±0.000	**0.002 ±0.000**
vehicle	0.004 ±0.000	0.007 ±0.008	0.065 ±0.051	0.097 ±0.086	0.069 ±0.059	0.006 ±0.007	0.064 ±0.050	0.018 ±0.000	**0.002 ±0.000**
vowel	0.003 ±0.000	0.012 ±0.011	0.084 ±0.023	0.075 ±0.028	0.046 ±0.021	0.013 ±0.013	0.041 ±0.014	0.011 ±0.000	**0.002 ±0.000**
wine	**0.001 ±0.000**	0.001 ±0.001	0.007 ±0.007	0.006 ±0.007	0.007 ±0.009	**0.001 ±0.000**	0.002 ±0.001	0.003 ±0.000	**0.001 ±0.000**
wisconsin	0.002 ±0.000	0.007 ±0.011	0.016 ±0.012	0.027 ±0.018	0.019 ±0.014	0.004 ±0.005	0.007 ±0.006	0.005 ±0.000	**0.001 ±0.000**
cold	**0.011 ±0.001**	0.033 ±0.021	0.071 ±0.073	0.096 ±0.086	0.122 ±0.165	0.048 ±0.053	0.128 ±0.095	0.099 ±0.007	0.017 ±0.005
diau	0.012 ±0.001	0.023 ±0.014	0.047 ±0.041	0.042 ±0.028	0.034 ±0.018	0.031 ±0.015	0.045 ±0.034	0.148 ±0.017	**0.011 ±0.002**
dtt	**0.010 ±0.001**	0.034 ±0.027	0.083 ±0.078	0.129 ±0.136	0.039 ±0.028	0.033 ±0.024	0.076 ±0.070	0.121 ±0.008	0.011 ±0.001
heat	0.012 ±0.001	0.025 ±0.016	0.054 ±0.039	0.097 ±0.141	0.102 ±0.143	0.036 ±0.035	0.065 ±0.069	0.101 ±0.006	**0.006 ±0.001**
spo	0.014 ±0.001	0.022 ±0.013	0.032 ±0.022	0.043 ±0.018	0.033 ±0.012	0.036 ±0.028	0.028 ±0.014	0.310 ±0.189	**0.006 ±0.001**

## Data Availability

The running examples of the paper together with other basic models are available at: https://github.com/alfaro96/scikit-lr (accessed on 30 March 2021).
